# Comparison of clinical outcome variables in patients with and without etomidate-facilitated anesthesia induction ahead of major cardiac surgery: a retrospective analysis

**DOI:** 10.1186/cc13988

**Published:** 2014-07-11

**Authors:** Sebastian Heinrich, Joachim Schmidt, Andreas Ackermann, Andreas Moritz, Frank Harig, Ixchel Castellanos

**Affiliations:** 1 Department of Anesthesiology, University Hospital Erlangen, Friedrich-Alexander-Universität Erlangen-Nürnberg, Krankenhausstr. 12, Erlangen 91054, Germany; 2 Department of Cardiac Surgery, University Hospital Erlangen, Ulmenweg 18, Erlangen 91054, Germany

## Abstract

**Introduction:**

It is well known that etomidate may cause adrenal insufficiency. However, the clinical relevance of adrenal suppression after a single dose of etomidate remains vague. The aim of this study was to investigate the association between the administration of a single dose of etomidate or an alternative induction regime ahead of major cardiac surgery and clinical outcome parameters associated with adrenal suppression and onset of sepsis.

**Methods:**

The anesthesia and intensive care unit (ICU) records from patients undergoing cardiac surgery over five consecutive years (2008 to 2012) were retrospectively analyzed. The focus of the analysis was on clinical parameters like mortality, ventilation hours, renal failure, and sepsis-linked serum parameters. Multivariate analysis and Cox regression were applied to derive the results.

**Results:**

In total, 3,054 patient records were analyzed. A group of 1,775 (58%) patients received a single dose of etomidate; 1,279 (42%) patients did not receive etomidate at any time. There was no difference in distribution of age, American Society of Anesthesiologists physical score, duration of surgery, and Acute Physiology and Chronic Health Evaluation II score. Postoperative data showed no significant differences between the two groups in regard to mortality (6.8% versus 6.4%), mean of mechanical ventilation hours (21.2 versus 19.7), days in the ICU (2.6 versus 2.5), hospital days (18.7 versus 17.4), sepsis-associated parameters, Sequential Organ Failure Assessment score, and incidence of renal failure. Administration of etomidate showed no significant influence (*P* = 0.6) on hospital mortality in the multivariate Cox analysis.

**Conclusions:**

This study found no evidence for differences in key clinical outcome parameters based on anesthesia induction with or without administration of a single dose of etomidate. In consequence, etomidate might remain an acceptable option for single-dose anesthesia induction.

## Introduction

Etomidate is considered an almost ideal induction agent because of the rapid onset and the predictable short duration of action in combination with minimal implications for the cardiovascular and pulmonary system [1]. These effects and benefits are even more important in high-risk cardiac surgery patients. However, soon after licensing of etomidate for long-term sedation and anesthesia in 1972, an increased mortality in intensive care patients resulted in recommendations to stop long-term use [2-4]. Even a single dose of etomidate inhibits transiently adrenal mitochondrial 11-β-hydroxylase activity with consecutive adrenal suppression [5,6]. Ever since, intensive care, emergency, and anesthesia communities have been discussing the use of even a single dose of etomidate controversially. Various studies and meta-analyses found either an increased [7-10] or an equal [11-17] risk of mortality and risk variables after administration of etomidate in critically ill patients with sepsis. In trauma patients, an increased susceptibility to pneumonia is attributed to etomidate [18].

Etomidate is frequently used for anesthesia induction ahead of cardiac surgery. In this context, smaller studies showed that etomidate is associated with increased vasopressor requirements [19]. Other authors report a relative adrenal insufficiency and deny an increase of vasopressor requirements [20]. In a retrospective analysis of 3,127 patients receiving etomidate or propofol, Wagner and colleagues [21] showed no increase in severe hypotension, in-hospital mortality, duration of hospital stay, intensive care unit (ICU) stay, and duration of mechanical ventilation. Further evidence for postoperative systemic inflammatory response syndrome (SIRS) due to adrenal insufficiency is of particular relevance for patients after on-pump cardiac surgery. It is well known that on-pump cardiac surgery per se is a risk factor for postoperative SIRS and infections [22]. The objective of our study was to determine differences in key clinical outcome parameters following anesthesia induction with administration of etomidate or an alternative induction regime. Analyzed parameters for clinical outcome were sepsis-associated serum parameters, duration of mechanical ventilation, morbidity scores, renal failure, length of ICU stay, and ICU and hospital mortality.

## Materials and methods

Ahead of this study, the local ethics committee (Ethics Committee of Friedrich-Alexander University Erlangen-Nürnberg) confirmed that no further patients’ consent and institutional ethical approval were required. This decision was based on the retrospective, descriptive, and anonymous study design and the fully closed patient files.

**Figure 1:**
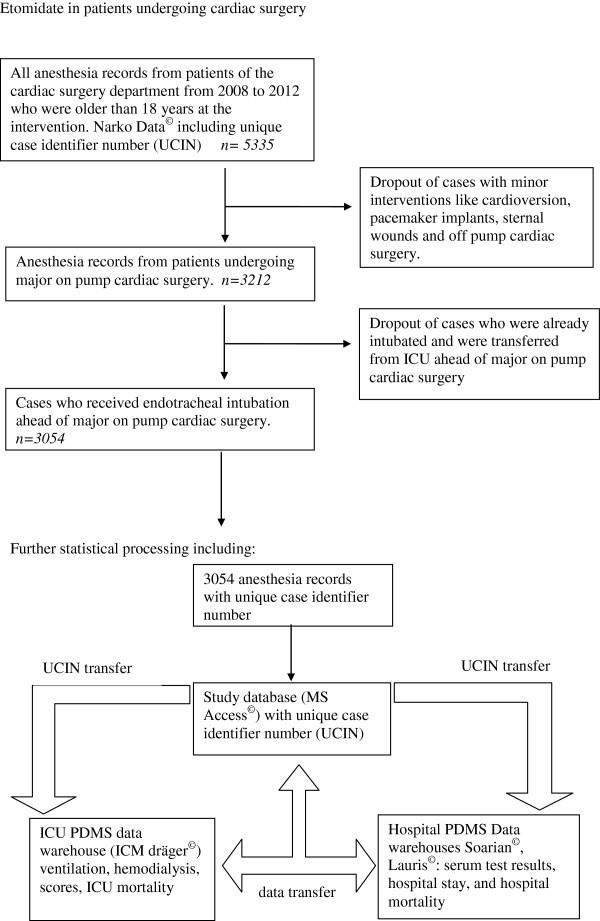
**Flow chart showing the case selection and data collection of the study.** ICU, intensive care unit; PDMS, patient data management system.

All anesthesia and ICU records from adult patients undergoing general anesthesia for major on-pump cardiac surgery at the university hospital in five consecutive years (January 2008 to December 2012) were analyzed retrospectively. The filed anesthesia records were retrieved from an electronic patient data management system (PDMS) (NarkoData Imeso, Hüttenberg, Germany) including an unambiguous and unique case identifier number (UCIN) (Figure 1). The UCIN is a pre-existing code of 13 digits to uniquely identify an individual case and is used to collect all available data from different PDMSs of a specific case to create the invoice for health insurance companies. Anonymized data were transferred to an MS Office 2010 Access® database (Microsoft Corporation, Redmond, WA, USA) including age, height, weight, Mallampati score, Acute Physiology and Chronic Health Evaluation II (APACHE II) score [23], Cormack and Lehane (CML) classification, priority of surgery, anesthesia drugs, and aortic clamping, bypass, and operation times. The postoperative ICU patient data were retrieved from the PDMS (ICM Dräger, Lübeck, Germany) and matched with the anesthesia data by using the UCIN. These data include laboratory parameters, duration of mechanical ventilation, length of ICU stay, ICU mortality, and Sequential Organ Failure Assessment (SOFA) score [24,25]. The ICM data are redundantly stored in the hospital PDMSs (Lauris and Soarian; Siemens, Erlangen, Germany) to facilitate access also from the normal care wards to these data (laboratory test results). Additional data which are available only in the hospital PDMSs (Lauris® and Soarian®) were also transferred and matched with the study database by using the UCIN. The data abstractors were qualified and experienced in the field of medical data management. For every PDMS, a single abstractor worked independently and fully blinded to the purpose of the study. The complete data of an observed case could be seen by the investigator group only after the matching in the study database. After the matching with the UCIN, the date of birth of the patient in the two datasets was compared to get a secondary check for the correct match of the datasets. If the date of birth had not been identical in the datasets, the study database would have alerted the investigator to a data mismatch (plausibility control). However, such a mismatch was not observed in any of the cases of this study.

Patients who reached the operation theatre already intubated and sedated were excluded from the analysis, as the anesthetic drugs used for intubation could not be verified in these cases. Patients with an ICU admission ahead of major cardiac surgery were also excluded from the analysis as their morbidity and mortality odds ahead of cardiac surgery may differ significantly from those of the other patients. For the same reason, cases with repetitive major cardiac surgery and off-pump coronary artery bypass grafting were excluded.

The cardiac surgery department contributing to this study provides a full spectrum of cardiac surgery for patients of every age, including heart transplantation and device surgery. The university hospital at Erlangen is a tertiary center with approximately 1,400 beds and provides about 900 on-pump cardiac surgery interventions per year, including around 250 interventions in pediatric cardiac surgery. In the cardiac surgery department, anesthetists have at least three years of professional experience and are under close supervision of senior physicians. Preoperative evaluation, induction, and maintenance of anesthesia as well as postoperative ICU treatment are performed in accordance with standard operating procedures (SOPs). The SOP for preoperative evaluation includes the documentation of the four-scale Mallampati test result [26]. Documentation of physical health status in accordance with the American Society of Anesthesiologists (ASA) was mandatory.

SOPs for anesthesia induction and maintenance demand a consistent approach, including arterial catheterization with local anesthesia, intravenous application of an opioid, a hypnotic drug, and a neuromuscular blocking agent. However, every anesthesiologist was free to vary the anesthetic drug regime for induction and maintenance. Etomidate (0.25 to 0.35 mg/kg) was the most commonly used hypnotic drug in this analysis. Alternatively, a combination of 1.5 to 2.0 mg/kg esketamine and 0.5 to 2 mg/kg propofol was administered for anesthesia induction. Cis-atracurium (0.15 to 0.2 mg/kg) was administered as standard neuromuscular blocking agent. In the case of a rapid sequence induction (RSI), 0.6 to 0.9 mg/kg rocuronium was given. The direct laryngeal view was graded in accordance with the four-scale CML classification [27]. Anesthesia was maintained with either continuous injection of propofol or inhalative application of sevoflurane which was also administered, in parts, in the pump circuit during cardiopulmonary bypass time. Continuous infusion of 0.4 to 0.8 μg/kg per hour sufentanil was the standard analgesic regime. All patients received continuous infusion of propofol at the end of surgery for transfer to the ICU and ongoing ICU sedation. Antimicrobial prophylaxis was performed by administration of a cephalosporine after the anesthesia induction and a repetitive dose after cardiopulmonary bypass. The antimicrobial regime remained unchanged during the study period. Admission SOP in the ICU for cardiac surgery patients includes hemogram and serum test of myocardial necrosis indicators like general creatinkinasis (CK), specific myocardial creatinkinasis (CK-MB), and troponin I (TROP) as well as inflammatory parameters like C-reactive protein (CRP). Procalcitonine (PCT) is not part of the standard blood testing but could be chosen by the attending physician for early detection of bacterial infection. The duration of mechanical ventilation was measured from ICU admission until extubation. Mechanical ventilation hours were summarized in cases of repetitive mechanical ventilation periods. SOFA score was obtained only when patients stayed more than 24 hours in the ICU.

### Statistics

Statistical processing was performed by using SPSS® 20.0 (IBM, Armonk, NY, USA; significance level *P* <0.05). Gaussian distribution of continuous variables was tested by using the Kolmogorov-Smirnov test. If continuous variables show Gaussian distribution, they are expressed as mean value and standard error and were compared by using the two-tailed Student *t* test. Categorical variables are given as absolute number and percentage of occurrence. A univariate analysis of categorical variables was performed by using the chi-squared test. Multivariate analysis for categorical variables and Cox analysis for survival variables was conducted where meaningful. An *ad posteriori* sample size calculation with estimated mortality rates of 3% and 5%, a statistical power of 0.8, and a confidence interval of 95% revealed a required number of at least 1,506 cases to enclose.

## Results

The definitive analysis includes a total of 3,054 out of 3,212 available cases, indicating a dropout rate of 5%. A group of 1,775 (58%) patients received etomidate ahead of cardiac surgery to facilitate anesthesia induction and endotracheal intubation. A group of 1,279 patients (42%) did not receive etomidate. The underlying demographic and preoperative data are shown in Table 1. There is a significantly higher number of female patients in the etomidate group: 554 (31%) versus 340 (27%) (*P* = 0.006). All other preoperative and demographic items show no significant difference.

**Table 1:** Preoperative and demographic data of the two study groups

**Categories**	**Non-etomidate group**	**Etomidate group**	***P*** **value**
Number of patients	1,279	1,775	
Sex			
Female	340 (27%)	554 (31%)	0.006
Male	939 (73%)	1,221 (69%)	
Age, years	67.9 ± 0.3	67.8 ± 0.2	0.600
Body mass index, kg/m^2^	28.2 ± 0.2 (24.8/30.7)	25.5 ± 0.2	0.994
ASA score			
1	3 (0.2%)	2 (0.1%)	0.413
2	31 (2%)	59 (3%)	0.147
3	1,143 (89%)	1,548 (87%)	0.069
4	101 (8%)	163 (9%)	0.212
5	1 (0.1%)	3 (0.2%)	0.494
APACHE II score			
	17.0 ± 0.2	16.7 ± 0.1	0.151
Missing	73 (5.7%)	101 (5.7%)	
Mallampati			
1	142 (11%)	246 (14%)	0.024
2	884 (69%)	1,139 (64%)	0.004
3	168 (13%)	307 (17%)	0.002
4	15 (1%)	16 (1%)	0.460
Missing	70 (6%)	67 (4%)	0.025
Pre-operative hemoglobin			
g/dL	13,1 ± 0.6	13.0 ± 0.5	0.899
Missing	217 (17%)	337 (19%)	
Pre-operative creatinine			
mg/dL	1.15 ± 0.2	1.15 ± 0.2	0.520
Missing	385 (30%)	365 (21%)	
Pre-operative creatinine kinase			
U/L	119.1 ± 5.5	125.2 ± 5.7	0.055
Missing	483 (38%)	487 (27%)	

Continuous variables are given as mean ± standard error, and categorical variables are presented as number (percentage). APACHE II, Acute Physiology and Chronic Health Evaluation II; ASA, American Society of Anesthesiologists.

Intraoperative data are shown in Table 2. The rate of poor laryngoscopic view given as CML 3 or 4 in the etomidate group is significantly lower than in the non-etomidate group (4.5% versus 9.8%; *P* <0.001). The rate of emergency cardiac surgery is significantly higher in the etomidate group. All other intraoperative items show no significant difference. A multivariate analysis revealed male gender, the absence of etomidate, and high Mallampati score as independent risk factors for poor laryngoscopic view given as CML 3 or 4 (Table 3).

**Table 2:** Intraoperative data of the two study groups

**Categories**	**Non-etomidate group**	**Etomidate group**	***P*** **value**
Number of patients	1,279	1,775	
Priority of surgery			
Elective	1,176 (92%)	1,562 (88%)	<0.001
Emergency	103 (8%)	213 (12%)	
Cormack-Lehane			
1	681 (53%)	1,334 (75%)	<0.001
2	466 (36%)	353 (20%)	<0.001
3	114 (9%)	71 (4%)	<0.001
4	12 (1%)	9 (0.5%)	0.154
Missing	4 (1%)	5 (0.5%)	0.875
Neuromuscular blocking agent			
Cisatracurium	1,204 (94%)	1,572 (89%)	<0.001
Rocuronium	30 (2%)	182 (10%)	<0.001
Suxamethonium	9 (1%)	21 (1%)	0.185
Missing	36 (3%)	0 (0%)	<0.001
Cut-to-suture time, minutes	198.2 ± 1.7	202.2 ± 1.6	0.138
Bypass time, minutes	83.5 ± 1.0	87.1 ± 1.0	0.105
Aortic clamping, minutes	49.0 ± 0.7	51.6 ± 0.6	0.299
Mechanical ventilation, hours	19.69 ± 1.5	21.22 ± 1.3	0.355
Intraoperative epinephrine, mg	102.0 ± 20.1	77.1 ± 14.2	0.054
Intraoperative norepinephrine, mg	1,165.7 ± 108.8	1,178 ± 79.0	0.458

Continuous variables are given as mean ± standard error, and categorical variables are presented as number (percentage).

**Table 3:** Multivariate analysis to identify risk factors for poor laryngoscopic view

**Co-variable**	**Odds ratio (95%CI)**	***P*** **value**
Male gender	0.87 (0.48-1.25)	<0.001
ASA score of 3 or 4	−0.12 (−0.93-0.69)	0.770
MLP 3 or 4	1.34 (1.04-1.64)	<0.001
Emergency surgery	0.07 (−0.40-0.55)	0.760
Administration of etomidate	−0.89 (−1.17-0.59)	<0.001
Administration of rocuronium	0.04 (−0.59-0.67)	0.899

ASA, American Society of Anesthesiologists; CI, confidence interval; MLP, Mallampati Score.

The main results addressing mortality and ICU course are presented in Tables 4 and 5. ICU discharge rate at the first postoperative day is significantly higher in the non-etomidate group than in the etomidate group: 694 (56%) versus 883 (51%) (*P* = 0.013). An isolated view on second postoperative day reveals significantly higher discharge rate of the etomidate group: 297 (17%) versus 158 (13%) (*P* <0.001). Summation of postoperative days 1 and 2 shows that there is no difference between the two study groups: 852 (68%) versus 1,180 (68%) (*P* = 0.937). On subsequent postoperative days, there was no difference between the groups until the end of observation on day 5.

**Table 4:** Postoperative data of the two study groups

**Categories**	**Non-etomidate group**	**Etomidate group**	***P*** **value**
Number of patients	1,279	1,775	
CRP, mg/L			
Missing	140 (10.9%)	80 (4.5%)	
Day 1	76.9 ± 1.2	77.0 ± 1.0	0.924
Day 2	183.8 ± 2.6	194.1 ± 2.2	0.382
Day 3	205.9 ± 3.8	211.5 ± 3.4	0.316
Day 4	189.7 ± 4.9	187.9 ± 4.3	0.340
Day 5	166.6 ± 6.5	150.9 ± 4.9	0.306
PCT, ng/mL			
Missing	732 (57.2%)	963 (54.2%)	
Day 1	4.1 ± 0.4	5.6 ± 0.5	0.053
Day 2	8.4 ± 0.9	10.0 ± 1.1	0.090
Day 3	7.9 ± 0.9	8.8 ± 0.9	0.312
Day 4	6.3 ± 0.9	6.6 ± 0.9	0.673
Day 5	4.7 ± 0.6	5.7 ± 0.7	0.254
Leukocytes, 10^3^/μL			
Missing	73 (5.7%)	63 (3.5%)	
Day 1	12.8 ± 0.2	11.7 ± 0.1	0.061
Day 2	11.9 ± 0.2	11.7 ± 0.2	0.327
Day 3	11.9 ± 0.3	11.7 ± 0.3	0.899
Day 4	11.5 ± 0.4	11.3 ± 0.4	0.969
Day 5	11.7 ± 0.5	11.3 ± 0.4	0.776
CK			
U/L	1347 ± 71	1642 ± 86	0.002
Missing	390 (30.5%)	226 (12.7%)	
CK-MB			
U/L	10.1 ± 0.2	10.0 ± 0.2	0.801
Missing	444 (34.7%)	292 (16.4%)	
Troponin			
μg/L	9.8 ± 0.6	11.5 ± 0.5	0.004
Missing	391 (30.5%)	230 (12.9%)	
Hemodialysis			
N	8.8%	10.2%	0.190
Hours	105 ± 12	112 ± 10	0.861
SOFA score			
Missing	655 (51.2%)	884 (49.8%)	
Day 1	7.9 ± 0.1	7.9 ± 0.1	0.340
Day 2	7.2 ± 0.2	7.3 ± 0.1	0.122
Day 3	7.4 ± 0.2	7.3 ± 0.2	0.697
Day 4	8.3 ± 0.3	8.2 ± 0.2	0.859
Day 5	8.8 ± 0.3	8.4 ± 0.3	0.394
Hospital days	17.4 ± 0.6	18.7 (0.6%)	0.250
ICU days	2.5 ± 0.1	2.6 ± 0.1	0.857
ICU discharge			
Day 1	694 (56%)	883 (51%)	0.013
Day 2	158 (13%) (∑852; 68%)	297 (17%) (∑1180; 68%)	0.937
Day 3	151 (12%) (∑1003; 80%)	205 (12%) (∑1385; 80%)	0.795
Day 4	80 (6%) (∑1083; 87%)	115 (7%) (∑1500; 87%)	0.898
Day 5	39 (3%) (∑1122; 90%)	51 (3%) (∑1551; 90%)	0.776
ICU re-admission	70 (5.5%)	102 (5.8%)	0.746
7-day mortality	39 (3.0%)	64 (3.6%)	0.401
30-day mortality	66 (5.2%)	106 (6.0%)	0.337
Overall in hospital mortality	82 (6.4%)	121(6.8)	0.657

Continuous variables are given as mean ± standard error, and categorical variables are presented as number (percentage). CK, creatinkinasis; CML, Cormack and Lehane; CRP, C-reactive protein; ICU, intensive care unit; PCT, procalcitonine; SOFA, Sequential Organ Failure Assessment.

**Table 5:** Multivariate cox analysis with survival data

**Co-variables**	**Hazard ratio (95%CI)**	***P*** **value**
Administration of etomidate	1.1 (0.79-1.51)	0.602
Body mass index of more than 40 kg/m^2^	1.25 (0.76-2.01)	0.361
Age of more than 75 years	1.15 (0.83-1.59)	0.396
ASA score of 4 or 5	2.20 (1.55-3.15)	<0.001
APACHE II score of more than 25	1.11 (1.08-1.14)	<0.001
ICU stay of more than 3 days	0.54 (0.38-0.79)	0.001
Re-admission to the ICU	1.79 (1.21-2.64)	0.004
Renal failure, hemofiltration within ICU stay	11.99 (8.02-17.92)	<0.001

APACHE II, Acute Physiology and Chronic Health Evaluation II; ASA, American Society of Anesthesiologists; CI, confidence interval; ICU, intensive care unit.

APACHE score of more than 25 (hazard ratio (HR) 1.1; *P* <0.001), ASA score of 4 or 5 (HR 2.2; *P* <0.001), ICU re-admission (HR 1.8; *P* = 0.004), requirement of renal replacement therapy (HR 11.9; *P* <0.001), and an ICU stay of longer than 3 days (HR 0.5; *P* = 0.001) were identified as mortality-predicting variables in a multivariate Cox regression analysis. For the administration of etomidate, body mass index (BMI) of more than 40 kg/m^2^, age of more than 75 years, and gender Cox analysis showed no significant influence on mortality (Table 5).

## Discussion

Key clinical outcome variables in patients with and without etomidate-facilitated anesthesia induction ahead of major cardiac surgery were comparable and showed no statistically significant differences. The demographic and preoperative data of our cohort indicate a good comparability of the two study groups as there are no statistical differences in age, BMI, APACHE II score, ASA status distribution, and preoperative blood test results. The higher frequency of female patients in the etomidate group is statistically significant but clinically of limited relevance. Little is known about gender differences in postoperative outcome after major cardiac surgery. The literature describes female gender as a risk factor for poor outcome after cardiac surgery because women are usually much older than male patients when undergoing major cardiac surgery [28,29]. The statistically higher rate of etomidate use in patients undergoing emergency cardiac surgery is remarkable. This is most likely due to the fact that emergency patients are more frequently in unstable hemodynamic conditions and preoperative fastening could not be awaited, so that a rapid sequence induction demands a short-onset of the anesthetic drug. The higher rate of emergency procedures in the etomidate group might also be causative for the slight increase of preoperative CK in this group.

Multivariate analysis identified high Mallampati score and male gender as risk factors for poor direct laryngoscopy. Patients who received etomidate for anesthesia induction showed significantly better intubation conditions than patients with an alternative induction regime, which is also reported by a former study [30]. Vasopressor requirements, duration of surgery, and aortic clamping and bypass times showed no significant difference between the two study groups.

The hypothesis of this study was that anesthesia induction using etomidate might lead to poorer clinical outcome due to impaired immune response. This comes along with higher vulnerability for infections and consecutively increased inflammatory serum parameters like CRP, PCT, and leukocyte count. Statistical processing is focused on relative differences between the study groups instead of deviation from normal values in order to account for the inherent inflammation associated with on-pump cardiac surgery. A recently published study showed that PCT is reliable in predicting severity of multi-organ failure and outcome of systemic inflammatory response [31]. In our study, none of the inflammation blood parameters shows a significant difference between the etomidate and the non-etomidate groups in the 5-day course we observed. To the best of our knowledge, no former study has evaluated the effect of etomidate on CRP, PCT, and leukocytes in cardiac surgery patients before.

SOFA score is a reliable and approved sepsis-associated score to reflect multi-organ failure, severity, and outcome in patients with sepsis [24,32,33]. In the present dataset, this score was documented when patients stayed longer than 24 hours in the ICU. We observed the SOFA score for five consecutive days and found no significant difference on any day between the etomidate and the non-etomidate groups. Renal dysfunction and failure constitute one of the most common organ dysfunctions in patients with sepsis [34,35] and are also associated with major cardiac surgery [36]. In the present study, we analyzed the rate of patients requiring renal replacement therapy as well as the hemodialysis hours per patient with renal failure. Neither the rate of patients needing hemodialysis nor the duration of hemodialysis (if required) showed statistically different results in the etomidate and the non-etomidate group. If etomidate administration for anesthesia induction ahead of cardiac surgery would have impaired the clinical outcome, mechanical ventilation hours should also be increased in this group. However, we found no evidence that single-dose use of etomidate ahead of major cardiac surgery increases the duration of mechanical ventilation. Our results are in line with a comparable analysis by Wagner and colleagues in which a prolonged mechanical ventilation time after administration of etomidate in patients undergoing coronary bypass surgery was not reported [21]. Additionally, a single dose of etomidate in patients with sepsis did not lead to an increased mechanical ventilation time [13]. In summary, we found no evidence that a single dose of etomidate for induction of anesthesia increased the risk of postoperative mortality or morbidity.

In patients who already have sepsis, the discussion of whether single-dose etomidate increases mortality risk is controversial. Some retrospective analyses suggested an increased risk of mortality in septic patients who received etomidate for intubation [5,6,9,32]. In contrast to these reports, other studies deny an increased risk of mortality for septic patients who received etomidate [10,12-16]. It could be hypothesized that cardiac surgery patients with severe comorbidities and impaired ventricular function should more likely have etomidate-triggered adrenal insufficiency and consecutive poor outcome. However, in our cohort, we found no evidence that etomidate leads to increased early or late ICU mortality. Even the total in-hospital mortality showed no difference. Moreover, neither the total length of stay in the ICU nor the total hospital stay differs significantly between the etomidate and the non-etomidate group. Patients of the non-etomidate group show a significantly higher ICU discharge on the first postoperative day. However, on day 2, the discharge rate is already balanced and remains balanced until the end of observation on day 5. The higher rate of emergency interventions in the etomidate group might be causative for a lower discharge rate on postoperative day 1. This theory is supported by the significantly higher peak of CK in the etomidate group which might be higher after myocardial infarction and emergency cardiac surgery. In regard to re-admission to the ICU, our study found no evidence that etomidate given ahead of cardiac surgery leads to a higher ICU re-admission rate. Given all of the postoperative items, the etomidate group showed no statistically significant or clinically relevant poorer outcome in any of the observed variables.

However, our study has some limitations which are based mainly on the retrospective design of the analysis. Owing to missing data in the pre- and postoperative serum test results, these items could not be taken into account for the Cox analysis. The SOFA and APACHE II scores show missing data and were not taken into account for the Cox analysis. The SOFA score was documented only for patients with more than 24 hours in the ICU. Cases which were discharged in the morning after surgery lack of SOFA score. APACHE II score was obtained when preoperative data were available. Where patients were transferred from external wards, these datasets were not available and APACHE II score could not be obtained.

Owing to its retrospective character, the study lacks a randomization protocol. Although the selection of induction regime with or without etomidate was based on the personal preferences of the attending anesthesiologist, a high number of anesthesiologists (135) performing the anesthesia procedures of the study might preclude a selection bias.

## Conclusions

This study found no evidence for differences in key clinical outcome parameters based on anesthesia induction with or without administration of a single dose of etomidate. This holds particularly true for mortality, length of hospital stay, length of ICU stay, re-admission to the ICU, duration of mechanical ventilation, postoperative systemic inflammatory response or sepsis discriminated by inflammatory parameters, renal failure, or SOFA score. Moreover, multivariate analysis revealed better intubation conditions in the etomidate group. Despite the known and undisputable risk of adrenal suppression and taking the beneficial effects for hemodynamic unstable and cardiac impaired patients into account, etomidate should remain an acceptable option in clinical routine for anesthesia induction.

## Key messages

Single-dose etomidate ahead of major on-pump cardiac surgery

● is not associated with higher postoperative mortality.

● is not associated with an increased rate of postoperative infection discriminated by serum tests and morbidity scores.

● is not associated with a higher SOFA score or an increased rate of renal replacement therapy.

● does not change length of ICU or total hospital stay.

## Abbreviations

APACHE II: Acute Physiology and Chronic Health Evaluation II; ASA: American Society of Anesthesiologists; BMI: body mass index; CK: creatinkinasis; CML: Cormack and Lehane; CRP: C-reactive protein; HR: hazard ratio; ICU: intensive care unit; PCT: procalcitonine; PDMS: patient data management system; SIRS: systemic inflammatory response syndrome; SOFA: Sequential Organ Failure Assessment; SOP: standard operating procedure; UCIN: unique case identifier number.

## Competing interests

The authors declare that they have no competing interests.

## Authors’ contributions

SH helped to design the study, to program the study database, to perform the statistical processing of the data, and to interpret the results and wrote the manuscript. JS helped to design the study, to interpret the results, and to supervise manuscript writing. IC helped to program the study database, to transcribe data from PDMSs to the study database, to interpret the results, and to supervise manuscript writing. AA helped to transcribe data from PDMSs to the study database. FH and AM helped to perform the statistical processing of the data and to interpret the results. All authors read and approved the final manuscript.

## References

[B1] Budde AO Mets B Pro: etomidate is the ideal induction agent for a cardiac anesthetic J Cardiothorac Vasc Anesth 2013 27 180 183 10.1053/j.jvca.2012.08.024 23127694

[B2] Watkins J Potential hazards of prolonged anaesthesia with etomidate and althesin Lancet 1983 1 1434 1435 6134190 10.1016/s0140-6736(83)92374-7

[B3] Miranda DR Stoutenbeek CP Etomidate in the intensive care unit Lancet 1983 2 684 685 6136824 10.1016/s0140-6736(83)92563-1

[B4] Shehabi Y Bellomo R Mehta S Riker R Takala J Intensive care sedation: the past, present and the future Crit Care 2013 17 322 10.1186/cc12679 23758942 PMC3706847

[B5] Absalom A Pledger D Kong A Adrenocortical function in critically ill patients 24 h after a single dose of etomidate Anaesthesia 1999 54 861 867 10.1046/j.1365-2044.1999.01003.x 10460557

[B6] Hildreth AN Mejia VA Maxwell RA Smith PW Dart BW Barker DE Adrenal suppression following a single dose of etomidate for rapid sequence induction: a prospective randomized study J Trauma 2008 65 573 579 10.1097/TA.0b013e31818255e8 18784570

[B7] Chan CM Mitchell AL Shorr AF Etomidate is associated with mortality and adrenal insufficiency in sepsis: a meta-analysis* Crit Care Med 2012 40 2945 2953 10.1097/CCM.0b013e31825fec26 22971586

[B8] Cuthbertson BH Sprung CL Annane D Chevret S Garfield M Goodman S Laterre PF Vincent JL Freivogel K Reinhart K Singer M Payen D Weiss YG The effects of etomidate on adrenal responsiveness and mortality in patients with septic shock Intensive Care Med 2009 35 1868 1876 10.1007/s00134-009-1603-4 19652948

[B9] Albert SG Ariyan S Rather A The effect of etomidate on adrenal function in critical illness: a systematic review Intensive Care Med 2011 37 901 910 10.1007/s00134-011-2160-1 21373823

[B10] Sunshine JE Deem S Weiss NS Yanez ND Daniel S Keech K Brown M Treggiari MM Etomidate, adrenal function, and mortality in critically ill patients Respir Care 2013 58 639 646 22906838 10.4187/respcare.01956 PMC4126750

[B11] Hohl CM Kelly-Smith CH Yeung TC Sweet DD Doyle-Waters MM Schulzer M The effect of a bolus dose of etomidate on cortisol levels, mortality, and health services utilization: a systematic review Ann Emerg Med 2010 56 105 113 e105 10.1016/j.annemergmed.2010.01.030 20346542

[B12] Dmello D Taylor S O’Brien J Matuschak GM Outcomes of etomidate in severe sepsis and septic shock Chest 2010 138 1327 1332 10.1378/chest.10-0790 20651024

[B13] McPhee LC Badawi O Fraser GL Lerwick PA Riker RR Zuckerman IH Franey C Seder DB Single-dose etomidate is not associated with increased mortality in ICU patients with sepsis: analysis of a large electronic ICU database Crit Care Med 2013 41 774 783 10.1097/CCM.0b013e318274190d 23318491

[B14] Ray DC McKeown DW Effect of induction agent on vasopressor and steroid use, and outcome in patients with septic shock Crit Care 2007 11 R56 10.1186/cc5916 17506873 PMC2206408

[B15] Riche FC Boutron CM Valleur P Berton C Laisne MJ Launay JM Chappuis P Peynet J Vicaut E Payen D Cholley BP Adrenal response in patients with septic shock of abdominal origin: relationship to survival Intensive Care Med 2007 33 1761 1766 10.1007/s00134-007-0770-4 17618417

[B16] Tekwani KL Watts HF Sweis RT Rzechula KH Kulstad EB A comparison of the effects of etomidate and midazolam on hospital length of stay in patients with suspected sepsis: a prospective, randomized study Ann Emerg Med 2010 56 481 489 10.1016/j.annemergmed.2010.05.034 20828877

[B17] Jung B Clavieras N Nougaret S Molinari N Roquilly A Cisse M Carr J Chanques G Asehnoune K Jaber S Effects of etomidate on complications related to intubation and on mortality in septic shock patients treated with hydrocortisone: a propensity score analysis Crit Care 2012 16 R224 10.1186/cc11871 23171852 PMC3672604

[B18] Asehnoune K Mahe PJ Seguin P Jaber S Jung B Guitton C Chatel-Josse N Subileau A Tellier AC Masson F Renard B Malledant Y Lejus C Volteau C Sébille V Roquilly A Etomidate increases susceptibility to pneumonia in trauma patients Intensive Care Med 2012 38 1673 1682 10.1007/s00134-012-2619-8 22777514

[B19] Iribarren JL Jimenez JJ Hernandez D Lorenzo L Brouard M Milena A Mora ML Martinez R Relative adrenal insufficiency and hemodynamic status in cardiopulmonary bypass surgery patients. A prospective cohort study J Cardiothorac Surg 2010 5 26 10.1186/1749-8090-5-26 20403156 PMC2867788

[B20] Morel J Salard M Castelain C Bayon MC Lambert P Vola M Auboyer C Molliex S Haemodynamic consequences of etomidate administration in elective cardiac surgery: a randomized double-blinded study Br J Anaesth 2011 107 503 509 10.1093/bja/aer169 21685487

[B21] Wagner CE Bick JS Johnson D Ahmad R Han X Ehrenfeld JM Schildcrout JS Pretorius M Etomidate use and postoperative outcomes among cardiac surgery patients Anesthesiology 2014 120 579 589 10.1097/ALN.0000000000000087 24296761 PMC3944222

[B22] Chalk K Meisel C Spies C Volk T Thuenemann K Linneweber J Wernecke KD Sander M Dysfunction of alveolar macrophages after 3 cardiac surgery and postoperative pneumonia? - an 5 observational study Crit Care 2013 17 R285 10.1186/cc13148 24321282 PMC4056566

[B23] Knaus WA Draper EA Wagner DP Zimmerman JE APACHE II: a severity of disease classification system Crit Care Med 1985 13 818 829 10.1097/00003246-198510000-00009 3928249

[B24] Vincent JL Moreno R Takala J Willatts S De Mendonca A Bruining H Reinhart CK Suter PM Thijs LG The SOFA (Sepsis-related Organ Failure Assessment) score to describe organ dysfunction/failure. On behalf of the Working Group on Sepsis-Related Problems of the European Society of Intensive Care Medicine Intensive Care Med 1996 22 707 710 10.1007/BF01709751 8844239

[B25] Castellanos I Bürkle T Prokosch H-U Schüttler J Concept for the hospital wiede implementation of a patient data management system at a large clinical center - an interdisciplinary challenge Anaesth Intensivmed 2009 50 618 629

[B26] Mallampati SR Gatt SP Gugino LD Desai SP Waraksa B Freiberger D Liu PL A clinical sign to predict difficult tracheal intubation: a prospective study Can Anaesth Soc J 1985 32 429 434 10.1007/BF03011357 4027773

[B27] Cormack RS Cormack-Lehane classification revisited Br J Anaesth 2010 105 867 868 10.1093/bja/aeq324 21081683

[B28] Zwolinski R Jander S Ostrowski S Bartczak K Adamek Kosmider A Banys A Jaszewski R Early and long term coronary artery bypass grafting outcomes in patients under 45 years of age Kardiol Pol 2013 71 32 39 23348531

[B29] Martin LM Holmes SD Henry LL Schlauch KA Stone LE Roots A Hunt SL Ad N Health-related quality of life after coronary artery bypass grafting surgery and the role of gender Cardiovasc Revasc Med 2012 13 321 327 23084324 10.1016/j.carrev.2012.09.002

[B30] Zed PJ Abu-Laban RB Harrison DW Intubating conditions and hemodynamic effects of etomidate for rapid sequence intubation in the emergency department: an observational cohort study Acad Emerg Med 2006 13 378 383 10.1111/j.1553-2712.2006.tb00313.x 16531603

[B31] Anand D Das S Ray S Bhargava S Srivastava LM Interrelationship between procalcitonin and organ failure in sepsis Indian J Clin Biochem 2014 29 93 96 10.1007/s12291-013-0326-z 24478557 PMC3903936

[B32] Jabre P Combes X Lapostolle F Dhaouadi M Ricard-Hibon A Vivien B Bertrand L Beltramini A Gamand P Albizzati S Perdrizet D Lebail G Chollet-Xemard C Maxime V Brun-Buisson C Lefrant JY Bollaert PE Megarbane B Ricard JD Anguel N Vicaut E Adnet F KETASED Collaborative Study Group Etomidate versus ketamine for rapid sequence intubation in acutely ill patients: a multicentre randomised controlled trial Lancet 2009 374 293 300 10.1016/S0140-6736(09)60949-1 19573904

[B33] Moreno R Vincent JL Matos R Mendonca A Cantraine F Thijs L Takala J Sprung C Antonelli M Bruining H Willatts S The use of maximum SOFA score to quantify organ dysfunction/failure in intensive care. Results of a prospective, multicentre study. Working Group on Sepsis related Problems of the ESICM Intensive Care Med 1999 25 686 696 10.1007/s001340050931 10470572

[B34] Guirgis FW Khadpe JD Kuntz GM Wears RL Kalynych CJ Jones AE Persistent organ dysfunction after severe sepsis: A systematic review J Crit Care 2014 29 320 326 10.1016/j.jcrc.2013.10.020 24360598

[B35] Gomez H Ince C De Backer D Pickkers P Payen D Hotchkiss J Kellum JA A unified theory of sepsis-induced acute kidney injury: inflammation, microcirculatory dysfunction, bioenergetics, and the tubular cell adaptation to injury Shock 2014 41 3 11 24346647 10.1097/SHK.0000000000000052 PMC3918942

[B36] Kiessling AH Dietz J Reyher C Stock UA Beiras-Fernandez A Moritz A Early postoperative serum cystatin C predicts severe acute kidney injury following cardiac surgery: a post-hoc analysis of a randomized controlled trial J Cardiothorac Surg 2014 9 10 10.1186/1749-8090-9-10 24397879 PMC3896845

